# Differences of virulence factors, and antimicrobial susceptibility according to phylogenetic group in uropathogenic *Escherichia coli* strains isolated from Korean patients

**DOI:** 10.1186/s12941-021-00481-4

**Published:** 2021-11-10

**Authors:** Miri Hyun, Ji Yeon Lee, Hyun ah Kim

**Affiliations:** 1 Department of Infectious Diseases, Keimyung University School of Medicine, 1095 Dalgubeol-daero, Dalseo-gu, Daegu, 42601 Republic of Korea; 2 Institute for Medical Science, Keimyung University, Daegu, Korea

**Keywords:** Urinary tract infection, *Escherichia coli*, Phylogenetic group, Virulence factor, Antimicrobial susceptibility

## Abstract

**Background:**

*Escherichia coli* is among the most common uropathogens. Increased antibiotic resistance in Gram negative bacilli is global concern. Alternative therapeutic options including vaccines against uropathogenic *E. coli* (UPEC) have been developed. In this study, we compared the genotypic characteristics and antimicrobial susceptibility of UPEC according to phylogenetic groups.

**Methods:**

We retrospectively reviewed the medical records of pyelonephritis patients with UPEC between February 2015 and June 2018. The study was conducted at a medical center in Korea. We compared the clinical and genotypic characteristics of UPEC according to phylogenetic groups. The phylogenetic groups and 29 virulence factors were identified using multiplex polymerase chain reaction.

**Results:**

Phylogenetic group analysis revealed that most uropathogenic *E. coli* belonged to groups B2 and D: B2 (276, 77.7%), D (62, 17.5%), B1 (12, 3.4%), and A (5, 1.4%). Among the virulence factors, *fyuA, fimH, traT, iutA, papG allele II,* and *papC* were the most frequently observed. Phylogenetic group B2 was more closely related to virulence factors, including *fimH, sfa/focED, focG, hlyA, cnf1, fyuA*, and *PAI*, than group D*.* Groups B2 and D showed similar clinical presentations and complications. Group B2 had mostly healthcare-associated infections and antimicrobial resistance. Group D mostly had community-acquired infections. The K1 serotype was prevalent in group B2, and K5 was the most prevalent in group D.

**Conclusions:**

Phylogenetic group B2 had more proportions and types of virulence factors than group D. Group B2 showed a high presentation of virulence factors related to adhesions and toxins. An increased presentation of antimicrobial resistance and healthcare-associated infections was also noted. Considering the genetic characteristics of UPEC, alternative therapeutic options targeting frequent virulence factors might be considered in addition to antibiotics.

## Background

Urinary tract infection (UTI) is one of the most common bacterial infections worldwide [1]. Among the uropathogens, uropathogenic *Escherichia coli* is the most predominant, causing up to 95% of community-acquired UTIs and 50% of healthcare-associated UTIs [2–4]. The clinical spectrum of UTI ranges from asymptomatic bacteriuria to cystitis, pyelonephritis, and prostatitis, and septic shock [5]. Clinical manifestations of UTI may differ depending on the underlying disease, preceding factors, and infecting bacteria [6, 7]. These manifestations can be influenced by bacterial pathogenicity [8]. In-hospital mortality is more dependent on uropathogens [4].

Increasing number of cases of multidrug-resistant gram-negative bacilli, and decreased efficacy of broad-spectrum antibiotics are global concerns [9, 10]. Ceftazidime/avibactam and ceftolozane/tazobactam, new alternative antibiotics of gram-negative bacilli, have been approved for clinical use; however, antimicrobial resistance to such antibiotics was reported even before the use of such antibiotics [11, 12]. Efforts are being made globally to develop alternative treatments or preventive methods. In addition to antibiotics, immunomodulatory agents, probiotics, and bacteriophages have been proposed as alternative therapeutic options for UTIs [13, 14]. Also, vaccines for uropathogenic *E. coli* is being developed to prevent recurrent UTI [15].

Uropathogenic *E. coli* have many virulence factors or properties, including adhesins, toxins, iron acquisition, and immune evasion that enable them to invade, colonize, and survive in the urinary tract [16]. Bacterial adhesion to uroepithelium is a crucial step of development of UTI [17]. The uropathogenic *E. coli* vaccine conducted up to the phase I study was ExPEC4V, and four types of *E. coli* O antigen were targeted [18]. Vaccines under development are targeting the virulence factors widely distributed in uropathogenic *E. coli* [16, 18–21]. Most vaccines for uropathogenic *E. coli* primarily target adhesion molecules, *iutA* and *fyuA* [19, 22]. *E. coli* can be categorized into four major groups, A, B1, B2, and D [23]. Among uropathogenic *E. coli*, phylogenetic group B2 is the most abundant, and the distributions vary from 33 to 80%. Phylogenetic group D was reported to account for about 10–30% [24–28]. The distribution of virulence factors may differ depending on the phylogenetic groups [20, 24]. Therefore, the analysis of virulence factors according to phylogenetic group B2 and D, which account for majority of uropathogenic *E. coli* cases, is important as basic data.

In this study, we compared virulence factors and antimicrobial susceptibility for the phylogenetic groups B2 and D and determined whether differences exist in clinical manifestations between the two groups. Additionally, the clinical characteristics and predisposing factors for the two groups were examined.

## Methods

### Study subjects

Patients who visited Keimyung University Dongsan Medical Center with uropathogenic *E. coli* UTI from February 2015 to June 2018 were categorized into phylogenetic group A, B1, B2, or D. Phylogenetic group B2 or D, which account for a large proportion of uropathogenic *E. coli*, were included in this study. Patients were divided into two groups according to phylogenetic group B2 and D. UTI was defined as a quantitative culture of ≥ 10^5^ CFU/mL for *E. coli* isolated from midstream urine or that collected using a catheter, and the presence of urinary symptoms such as urgency, high frequency of urination, and dysuria. Diagnostic criteria for upper UTI included fever, flank pain, urinary symptoms, and/or tenderness of the costovertebral angle. Patients aged < 18 years or with polymicrobial infections were excluded, along with patients transferred to other hospitals during their treatment period. If the causative bacterium of a patient with UTI was *E. coli*, it was defined as uropathogenic *E. coli*. *E. coli* isolates from the blood, urine, or pus were collected, and only one isolate per patient was examined. The categories of infection were further divided into community-acquired, healthcare-associated, and nosocomial infections. Community-acquired infections were defined as those in which symptoms occurred within 48 h after visiting the hospital. However, patients with community-acquired infections and healthcare-associated risk factors were categorized under healthcare-associated infections. Healthcare-associated risk factors included hospitalization within 90 days, hemodialysis, intravenous medication in outpatient clinics, or residency in long-term care facilities. Nosocomial infections were defined as those in which symptoms occurred 48 h after hospital admission. This study was approved by the Institutional Review Board of Keimyung University Dongsan Medical Center (File No. 2020-02-003). The requirement for written informed consent was waived by the committee because of the retrospective nature of the study and the use of identifiable specimens. Medical records were reviewed retrospectively.

### Study design

#### Data collection

Medical records, including underlying diseases, predisposing factors, antibiotics used within last 3 months, previous hospitalization, antimicrobial susceptibility, clinical features, current antibiotics being administered, and treatment outcomes, were retrospectively analyzed. Obstructive UTI was defined as UTI due to urinary tract obstruction such as one of the following: benign prostate hyperplasia, uterine prolapse, or malignancy. Urinary tract stones were not regarded as obstructive UTI and were classified as a predisposing factor. Severe UTI was defined as UTI combined with multiorgan failure or hypotension and complicated UTI as UTI with predisposing factors for persistent and relapsing infections, such as urinary tract stones, foreign bodies (for example, indwelling urinary catheters or other drainage devices), or obstructions. The short-term treatment outcome was determined after 72 h of empirical antibiotic treatment based on persistent fever and acute kidney injury. Persistent fever was defined as fever persisting over 72 h. Acute kidney injury was defined as an increase in serum creatinine level by > 0.3 mg/dL within 48 h, increase in serum creatinine level to > 1.5 times baseline within 7 days or increase in urine volume < 0.5 mL/kg/h for 6 h. The long-term outcome was determined by infection-related 30-day mortality and relapsed UTI within 3 months. Infection-related 30-day mortality was defined as death due to uropathogenic *E. coli* UTI or complications of infection within 30 days. McCabe-Jackson score was used as the criteria to predict the survival of patients with gram-negative bacteremia based on the level of the underlying diseases, which were classified as rapidly fatal disease, ultimately fatal disease, and nonfatal underlying disease [29].

#### Phylogenetic groups

Phylogenetic groups of the *E. coli* isolates were determined using the polymerase chain reaction (PCR)-based method developed by Doumith et al. [1]. *E. coli* were categorized into one of the four main phylogenetic groups—A, B1, B2, and D—using four phylogenetic group markers—*gadA, chuA, yjaA*, and TSPE4.C2. The groups were determined according to the different combinations of the four amplicons. Crude deoxyribonucleic acid (DNA) was prepared by lysis of colonies in 500 μL of sterile distilled water at 100 °C for 15 min, followed by centrifugation. The lysis supernatant was used for the polymerase chain reaction. The polymerase chain reaction conditions were as follows: an initial activation at 94 °C for 4 min; then, 30 cycles at 94 °C for 30 s, 65 °C for 30 s, 72 °C for 30 s; and finally, extension at 72 °C for 5 min [23]. The primers used in this study are listed in Table 1.

**Table 1:** Primers used for phylogenetic groups in this study

Marker	Primer direction	Primer sequence (5′-3′)	Product length (bp)
*gadA*	Forward Reverse	GATGAAATGGCGTTGGCGCAAG GGCGGAAGTCCCAGACGATATCC	373
*ChuA*	Forward Reverse	ATGATCATCGCGGCGTGCTG AAACGCGCTCGCGCCTAAT	281
*yjaA*	Forward Reverse	TGTTCGCGATCTTGAAAGCAAACGT ACCTGTGACAAACCGCCCTCA	216
TSPE4.C2	Forward Reverse	GCGGGTGAGACAGAAACGCG TTGTCGTGAGTTGCGAACCCG	152

#### Virulence genes

Virulence genes were detected using a multiplex polymerase chain reaction assay developed by Johnson and Stell [2]. This involved five primer pools, with 29 primers listed in order of decreasing amplicon size (bp) within each pool as follows: pool 1: *PAI, papA, fimH, kpsMT* III, *papEF*, and *ibeA*; pool 2: *fyuA, bmaE, sfa/focDE, iutA, papG allele* III, and K1; pool 3: *hlyA, rfc, nfaE, papG allele* I, *kpsMT* II, and *papC*; pool 4: *gafD, cvaC, cdtB, focG, traT*, and *papG allele* II; and pool 5: *papG allele* I, *papG alleles* II and III, *afa/draBC, cnf1, sfas*, and K5. The reaction was conducted with an initial activation at 95 °C for 12 min; followed by 25 cycles of denaturation (94 °C, 30 s), annealing (63 °C, 30 s), and extension (68 °C, 3 min); and a final extension at 72 °C for 10 min. The amplicons were electrophoresed in 2% agarose gels, stained with ethidium bromide, and destained with distilled water [30]. The primers used in this study are listed in Table 2.

**Table 2:** Primers used for virulence factors used in this study

Marker	Primer direction	Primer sequence (5′-3′)	Product length (bp)
*papA*	Forward Reverse	ATGGCAGTGGTGTCTTTTGGTG CGTCCCACCATACGTGCTCTTC	720
*papC*	Forward Reverse	GTGGCAGTATGAGTAATGACCGTTA ATATCCTTTCTGCAGGGATGCAATA	200
*papEF*	Forward Reverse	GCAACAGCAACGCTGGTTGCATCAT AGAGAGAGCCACTCTTATACGGACA	336
*papG allele* I	Forward Reverse	TCGTGCTCAGGTCCGGAATTT TGGCATCCCCCAACATTATCG	461
*papG allele* II	Forward Reverse	GGGATGAGCGGGCCTTTGAT CGGGCCCCCAAGTAACTCG	190
*papG allele* III	Forward Reverse	GGCCTGCAATGGATTTACCTGG CCACCAAATGACCATGCCAGAC	258
*sfa/focDE*	Forward Reverse	CTCCGGAGAACTGGGTGCATFTTAC CGGAGGAGTAATTACAAACCTGGCA	410
*sfaS*	Forward Reverse	GTGGATACGACGATTACTGTG CCGCCAGCATTCCCTGTATTC	240
*focG*	Forward Reverse	CAGCACAGGCAGTGGATACGA GAATGTCGCCTGCCCATTGCT	360
*afa/draBC*	Forward Reverse	GGCAGAGGGCCGGCAACAGGC CCCGTAACGCGCCAGCATCTC	559
*bmaE*	Forward Reverse	ATGGCGCTAACTTGCCATGCTG AGGGGGACATATAGCCCCCTTC	507
*gafD*	Forward Reverse	TGTTGGACCGTCTCAGGGCTC CTCCCGGAACTCGCTGTTACT	952
*nfaE*	Forward Reverse	GCTTACTGATTCTGGGATGGA CGGTGGCCGAGTCATATGCCA	559
*fimH*	Forward Reverse	TGCAGAACGGATAAGCCGTGG GCAGTCACCTGCCCTCCGGTA	508
*hlyA*	Forward Reverse	AACAAGGATAAGCACTGTTCTGGCT ACCATATAAGCGGTCATTCCCGTCA	1177
*cnf1*	Forward Reverse	AAGATGGAGTTTCCTATGCAGGAG CATTCAGAGTCCTGCCCTCATTATT	498
*fyuA*	Forward Reverse	TGATTAACCCCGCGACGGGAA CGCAGTAGGCACGATGTTGTA	880
*iutA*	Forward Reverse	GGCTGGACATCATGGGAACTGG CGTCGGGAACGGGTAGAATCG	300
*kpsMT* II	Forward Reverse	GCGCATTTGCTGATACTGTTG CATCCAGACGATAAGCATGAGCA	272
*kpsMT* III	Forward Reverse	TCCTCTTGCTACTATTCCCCCT AGGCGTATCCATCCCTCCTAAC	392
*rfc*	Forward Reverse	ATCCATCAGGAGGGGACTGGA AACCATACCAACCAATGCGAG	788
*ibeA*	Forward Reverse	AGGCAGGTGTGCGCCGCGTAC TGGTGCTCCGGCAAACCATGC	170
*cvaC*	Forward Reverse	CACACACAAACGGGAGCTGTT CTTCCCGCAGCATAGTTCCAT	680
*traT*	Forward Reverse	GGTGTGGTGCGATGAGCACAG CACGGTTCAGCCATCCCTGAG	290
*PAI*	Forward Reverse	GGACATCCTGTTACATCGCGCA TCGCCACCAATCACAGCCGAAC	930

*PAI* pathogenicity island

#### Antibiotic resistance and extended spectrum beta-lactamase (ESBL)-disk diffusion test

Clinical specimens, such as blood, urine, and pus, were collected for microbial identification. *E. coli* was isolated using a Vitek system (BioMerieux, Lyon, France). Antimicrobial susceptibility profiles were determined by interpreting the breakpoints recommended by the Clinical and Laboratory Standards Institute (CLSI) guideline of 2016 [31]. ESBL production was detected using automated methodology, namely, the Phoenix GN Combo Panels 448541, which were used to inoculate and incubate bacteria according to the manufacturer’s recommendations [32]. Disk diffusion test was also performed to double check the ESBL-producing strains. Disk diffusion tests were performed in cases of resistance to cefotaxime or ceftazidime, twice for each specimen, and interpreted according to the 2020 CLSI guidelines, using Mueller-Hinton agar. Thirty microgram disks containing ceftazidime and ceftriaxone and 30/10 μg disks containing cefotaxime/clavulanate or ceftazidime/clavulanate were used (BD BBL^TM^ Sensi-Disc^TM^ Antimicrobial Susceptibility Test Discs, BD Diagnostic Systems, Sparks, Maryland, U.S.A) [33].

### Statistical analysis

Statistical analysis was performed using the Statistical Package for the Social Sciences software (version 21.0; SPSS Inc., IBM Corp., Armonk, NY, USA). Categories were compared using the chi-square test or Fisher’s exact test. For continuous variables, the normal distribution was calculated using the Kolmogorov–Smirnov test. The Mann–Whitney *U* test and independent *t*-test were performed for data that followed non-normal and normal distributions, respectively. Statistical significance was defined as *P* < 0.05.

## Results

### Basic characteristics of the study group

Phylogenetic group analysis revealed that most uropathogenic *E. coli* belonged to groups B2 and D: B2 (276, 77.75%), D (62, 17.46%), B1 (12, 3.38%), and A (5, 1.41%). The clinical characteristics of phylogenetic group A, B1, B2, and D were briefly summarized in Table 3. Among the 4 phylogenetic groups, we compared group B2 and D in this study.

**Table 3:** Baseline characteristics of uropathogenic *Escherichia coli* infection according to phylogenetic group

	Phylogenetic group A (n = 5)	Phylogenetic group B1 (n = 12)	Phylogenetic group B2 (n = 276)	Phylogenetic group D (n = 62)
Age, years	67 (59–84)	74 (69–78)	69.43 ± 14.59	69.16 ± 14.13
Male sex	2 (40.0%)	1 (8.3%)	57 (20.7%)	4 (6.5%)
Category of UTI				
Acute pyelonephritis	5 (100.0%)	12 (100.0%)	269 (97.5%)	61 (98.4%)
Acute prostatitis	0 (0.0%)	0 (0.0%)	8 (2.9%)	2 (3.2%)
Renal abscess	0 (0.0%)	0 (0.0%)	20 (7.2%)	8 (12.9%)
Prostatic abscess	0 (0.0%)	0 (0.0%)	4 (1.4%)	0 (0.0%)
Category of infection				
Community-acquired	4 (80.0%)	8 (66.7%)	192 (69.6%)	52 (83.9%)
Healthcare-associated	1 (20.0%)	4 (33.3%)	71 (25.7%)	7 (11.3%)
Nosocomial	0 (0.0%)	0 (0.0%)	13 (4.7%)	3 (4.8%)

In group B2, 276 patients were included; 57 (20.7%) were men, and the mean age was 69.43 years. In group D, 62 patients were included; 4 (6.5%) were men, and the mean age was 69.16 years. The proportion of male patients was significantly higher in the group B2 than in other groups (*P* = 0.009). For the underlying diseases, diabetes mellitus (DM) was more frequently observed in group D (51.6%) than in group B2 (36.6%) (*P* = 0.029). No significant differences with respect to underlying diseases were observed between the two groups, except for DM. The McCabe-Jackson score indicated no significant differences between the two groups. Obstructive uropathy and previous use of urinary catheters were more frequently observed in group B2 than in other groups, but without significant difference. Complicated UTI was more frequently observed in group B2 than in other groups (*P* = 0.009). Bacteremic UTI and severe UTIs did not differ significantly between the two groups; besides, analysis of UTI categories revealed no significant difference in the proportions of renal abscess, acute prostatitis, and prostatic abscess. However, analysis of infection categories revealed that the prevalence of community-acquired and healthcare-associated infections were significantly higher in groups D and B2, respectively, than in other groups (Table 4).

**Table 4:** Baseline characteristics and clinical manifestations of uropathogenic *Escherichia coli* infection according to phylogenetic group

	Phylogenetic group B2 (n = 276)	Phylogenetic group D (n = 62)	*p* value
Age, years	69.43 ± 14.59	69.16 ± 14.13	0.893
Male sex	57 (20.7%)	4 (6.5%)	0.009
Category of UTI			
Acute pyelonephritis	269 (97.5%)	61 (98.4%)	0.999*
Acute prostatitis	8 (2.9%)	2 (3.2%)	0.999*
Renal abscess	20 (7.2%)	8 (12.9%)	0.144
Prostatic abscess	4 (1.4%)	0 (0.0%)	0.999*
Category of infection			
Community-acquired	192 (69.6%)	52 (83.9%)	0.023
Healthcare-associated	71 (25.7%)	7 (11.3%)	0.015
Nosocomial	13 (4.7%)	3 (4.8%)	0.999*
Underlying diseases			
Solid tumor	39 (14.1%)	9 (14.5%)	0.937
Hematologic malignancy	0	0	
Chronic liver disease	43 (15.6%)	7 (11.3%)	0.390
Liver cirrhosis	14 (5.1%)	0 (0.0%)	0.082*
Cardiovascular disease	75 (27.2%)	16 (25.8%)	0.826
Hypertension	143 (51.8%)	39 (62.9%)	0.113
Neurologic disease	98 (35.5%)	19 (30.6%)	0.467
Chronic renal disease	14 (5.1%)	4 (6.5%)	0.753*
Diabetes mellitus	101 (36.6%)	32 (51.6%)	0.029
Chronic lung disease	28 (10.1%)	7 (11.3%)	0.789
Solid organ transplantation	2 (0.7%)	0 (0.0%)	0.999*
Predisposing factors			
Pregnancy	1 (0.4%)	0 (0.0%)	0.999*
Neurogenic bladder	24 (8.7%)	5 (8.1%)	0.873
BPH or uterine prolapse	26 (9.4%)	2 (3.2%)	0.110
Urogenic anomaly	4 (1.4%)	0 (0.0%)	0.999*
Nephrectomy state (one kidney)	3 (1.1%)	1 (1.6%)	0.557*
Neutropenia	0	0	
Previous genitourinary surgery or procedure within 72 h	0	0	
Recurrent UTI	32 (11.6%)	6 (9.7%)	0.666
Presence of urologic devices	1 (0.4%)	0 (0.0%)	0.999*
Intermittent catheterization	2 (0.7%)	0 (0.0%)	0.999*
Urinary catheter	23 (8.3%)	3 (4.8%)	0.439*
Prior antibiotics within 3 months	64 (23.2%)	12 (19.4%)	0.514
Type of UTI			
Bacteremic UTI	167 (60.5%)	44 (71.0%)	0.124
Complicated UTI	57 (20.7%)	4 (6.5%)	0.009
Severe UTI	94 (34.1%)	21 (33.9%)	0.978

*BPH* benign prostate hyperplasia, *UTI* urinary tract infection

*Fisher’s extract test

### Comparison of virulence factors between phylogenetic groups B2 and D

*FimH* and *fyuA* were the most common virulence factors in both groups. Adhesion molecules were identified in both groups, and their distribution was similar. *FimH* (99.6% vs. 90.3%, *P* < 0.001)*, sfa/focED* (17.0% vs. 0.0%, *P* < 0.001)*,* and *focG* (12.3% vs. 3.2%, *P* = 0.036) were more common in phylogenetic group B2 than in D. Phylogenetic group B2 was the most closely related to virulence factors associated with adhesion, toxins, iron metabolism, and *PAI*. Group B2 had higher levels of toxin-associated virulence: *hlyA* (phylogenetic group B2 = 33.3% vs. D = 6.5%, *P* < 0.001), *cnf1* (39.9% vs. 0.0%, *P* < 0.001), and *cvaC* (8.7% vs. 0.0%, *P* = 0.011); and iron metabolism-associated virulence factors: *fyuA* (99.6% vs. 93.5%, *P* = 0.004); and *PAI* (88.8% vs. 19.4%, *P* < 0.001) than group D. With regard to protection molecules, no significant differences were observed between the two groups. The K1 serotype was prevalent in the phylogenetic group B2, whereas K5 was widespread in group D (Table 5).

**Table 5:** Virulence factors of uropathogenic *Escherichia coli* classified by phylogenetic group

	Phylogenetic group B2 (n = 276)	Phylogenetic group D (n = 62)	*p* value
Adhesion molecule			
* papA*	186 (67.4%)	41 (66.1%)	0.848
* papEF*	40 (14.5%)	7 (11.3%)	0.510
* papC*	195 (70.7%)	43 (69.4%)	0.840
* papG*	133 (48.2%)	28 (45.2%)	0.666
* papG allele I*	1 (0.4%)	0 (0.0%)	0.999*
* papG allele II*	196 (71.0%)	46 (74.2%)	0.616
* papG allele III*	7 (2.5%)	0 (0.0%)	0.357*
* fimH*	275 (99.6%)	56 (90.3%)	< 0.001*
* afa/draBC*	38 (13.8%)	12 (19.4%)	0.263
* sfaS*	15 (5.4%)	5 (8.1%)	0.385*
* sfa/focED*	47 (17.0%)	0 (0.0%)	< 0.001
* bmaE*	1 (0.4%)	0 (0.0%)	0.999*
* gafD*	0	0	
* nfaE*	1 (0.4%)	2 (3.2%)	0.088*
* focG*	34 (12.3%)	2 (3.2%)	0.036
Toxin			
* hlyA*	92 (33.3%)	4 (6.5%)	< 0.001
* cnf1*	110 (39.9%)	0 (0.0%)	< 0.001
* cvaC*	24 (8.7%)	0 (0.0%)	0.011*
* cdtB*	0	0	
Iron metabolism			
* fyuA*	275 (99.6%)	58 (93.5%)	0.004*
* iutA*	203 (73.6%)	47 (75.8%)	0.715
Protection, Capsule			
* kpsMT II*	159 (57.6%)	39 (62.9%)	0.444
* kpsMT III*	3 (1.1%)	2 (3.2%)	0.228*
* rfc*	4 (1.4%)	1 (1.6%)	0.999*
* traT*	214 (77.5%)	49 (79.0%)	0.798
Others			
* PAI*	245 (88.8%)	12 (19.4%)	< 0.001
* ibeA*	18 (6.5%)	1 (1.6%)	0.218*
* K1*	88 (31.9%)	3 (4.8%)	< 0.001
* K5*	60 (21.7%)	21 (33.9%)	0.043

*PAI* pathogenicity island

*Fisher’s extract test

### Comparison of antibiotic resistance, empirical antibiotics, and antibiotic adequacy

The rates of resistance to ciprofloxacin, cefotaxime, and trimethoprim/sulfamethoxazole were 50.5%, 45.1%, and 37.1% in group B2 and 22.6%, 29.0%, and 48.4% in group D (*P* < 0.001; *P* < 0.001; and *P* = 0.100, without significance difference). The proportions of ESBL-producing *E. coli* in Phoenix GN Combo Panels were 44.0% and 27.4% in groups B and D, respectively (*P* = 0.016): in the double-disk diffusion test, the proportions of ESBL-producing *E. coli* were 27.2% and 17.7% in groups B2 and D, respectively (*P* = 0.123) (Table 6). Among ESBL-producing *E. coli*, resistance rates to ciprofloxacin, piperacillin/tazobactam, and trimethoprim/sulfamethoxazole were 87.6%, 14.0%, and 56.2% in group B2 and 47.1%, 11.8%, and 58.8% in group D (*P* < 0.001; *P* = 0.999; and *P* = 0.838, without significance difference). For both groups, the most commonly used empirical antibiotic was ceftriaxone. Eighty-four (66.7%) and 48 cases (77.4%) in groups B2 and D, respectively, were evaluated to have used concordant initial antibiotics.

**Table 6:** Antibiotic resistance of uropathogenic *Escherichia coli* classified by phylogenetic group

	Phylogenetic group B2 (n = 276)	Phylogenetic group D (n = 62)	*p* value
Resistance			
Amikacin	2 (0.8%)	0 (0.0%)	0.999*
Amoxicillin/clavulanate	118 (42.9%)	9 (14.6%)	< 0.001
Ampicillin	210 (76.4%)	47 (75.8%)	0.926
Aztreonam	120 (43.6%)	17 (27.4%)	0.019
Cefazolin	134 (48.7%)	18 (29.0%)	0.005
Cefepime	120 (43.6%)	17 (27.4%)	0.019
Cefotaxime	124 (45.1%)	18 (29.0%)	0.022
Cefoxitin	25 (9.1%)	5 (8.1%)	0.804
Ceftazidime	121 (44.0%)	16 (25.8%)	0.008
Ciprofloxacin	139 (50.5%)	14 (22.6%)	< 0.001
Ertapenem	0	0	
Gentamicin	94 (34.2%)	17 (27.4%)	0.315
Imipenem	0	0	
Piperacillin/tazobactam	25 (9.1%)	3 (4.8%)	0.276
Tigecycline	0	0	
Trimethoprim/sulfamethoxazole	102 (37.1%)	30 (48.4%)	0.100
ESBL	121 (44.0%)	17 (27.4%)	0.016
ESBL double disk	75 (27.2%)	11 (17.7%)	0.123

*ESBL* extended-spectrum beta-lactamase

*Fisher’s extract test

### Comparison of treatment outcomes

In early outcomes, 22.8% of cases had persistent fever and 17.0% experienced acute kidney injury during the hospital stay for group B2; for D, 22.6% and 17.7% of cases experienced persistent fever and acute kidney injury, respectively. Differences in persistent fever and acute kidney injury were insignificant between the two groups. Duration of hospital stay, 30-day mortalities, and infection-related 30-day mortality were 14.90 days, 1.8%, and 0.7% in group B2 and 12.71 days, 1.6%, and 0.0% in group D (without significant difference; *P* = 0.999 and *P* = 0.999). Six and one patient died in groups B2 and D, respectively. After they were diagnosed with UTI, the median period from diagnosis to death in group B2 was 9.5 days (interquartile range 7.0–25.75 days), and in group D, a patient died on day 3. Within 3 months, UTI events relapsed in 7.6% and 8.1% of B2 and D members, respectively, which were not significantly different (Table 7).

**Table 7:** Outcomes of uropathogenic *Escherichia coli* infection classified by phylogenetic group

	Phylogenetic group B2 (n = 276)	Phylogenetic group D (n = 62)	*p* value
Persistent fever	63 (22.8%)	14 (22.6%)	0.967
Acute kidney injury	47 (17.0%)	11 (17.7%)	0.893
30-Day mortality	5 (1.8%)	1 (1.6%)	0.999*
Infection-related 30-day mortality	2 (0.7%)	0 (0.0%)	0.999*
Total duration of hospital stay, days	14.90 ± 10.70	12.71 ± 7.74	0.128
Time to death, days	9.50 (7.0–25.75)	3 (3–3)	0.313
Relapse within 3 months	21 (7.6%)	5 (8.1%)	0.999*

*Fisher’s extract test

## Discussion

In this study, the phylogenetic groups B2 and D exhibited different characteristics. Phylogenetic group B2 had more virulence factors, especially higher presentation of adhesion-related (S fimbriae, F fimbriae), toxin-related (hemolysin A, cytotoxic necrotizing factor 1), and iron metabolism-related virulence factors (*fyuA*), than group D. Greater antimicrobial resistance and healthcare-associated infection was also noted in group B2 than in group D. Phylogenetic group D was associated with community-acquired UTI and exhibited a lower association with virulence and predisposing factors than group B. No significant differences in clinical manifestations and treatment outcomes between the phylogenetic groups B2 and D occurred.

Pathogenic strains of *E. coli* have been classified by the identification of O, K, and H antigens [16]. A phylogenetic study revealed that *E. coli* can be separated into four major groups: A, B1, B2, and D [23] and are classified into three main groups according to genetic and clinical criteria: commensal, intestinal pathogenic, and extraintestinal pathogenic strains [16]. Among the extraintestinal pathogenic *E. coli*, some strains such as uropathogenic *E. coli* could survive in the gut and colonize the periurethral area, resulting in UTIs. The uropathogenic *E. coli*, which are known as the virulent strains, belong to the phylogenetic group B2 or D, and the less virulent strains mainly belong to A or B1 and are commensal strains [30].

The phylogenetic group of uropathogenic *E. coli* mainly comprised B2 group, but the distributions and proportions of phylogenetic groups and virulence factors vary based on the country where the study was conducted and study settings. In Italy, phylogenetic group B1 was the most prevalent in both community-acquired acute pyelonephritis and recurrent cystitis in females. The distribution and proportion of phylogenetic groups of acute pyelonephritis by uropathogenic *E. coli* were as follows: group B1, 68.7%; group A, 27.8%; and group D, 11.1%. Toxin-associated and siderophore-associated virulence factors were frequently observed in patients with recurrent cystitis [34]. In a study of community-acquired UTIs in Iran, phylogenetic group B2 was most frequently detected. The phylogenetic groups were as follows: group B2, 67.3%; group D, 21.4%; group A, 6.5%; and group B1, 4.8% [26]. In a study of community-acquired UTIs in Korea, phylogenetic group B2 was the most frequently detected, followed by groups D and A [24]. Previous reports were comparative studies of phylogenetic groups in community-acquired UTI. In Mexico, the phylogenetic groups of outpatient UTI were compared, and B2 (51.0%) was the most common, followed by A (13.4%), B1 (10.3%), and D (9.8%) in that order [27]. A Turkish study evaluated the distribution of phylogenetic groups in community-acquired UTI and nosocomial UTI, including cystitis and pyelonephritis. Phylogenetic group B2 was the most common in both community-acquired UTI and nosocomial UTI [25]. In a study of UTI in Mongolia, the proportion of phylogenetic groups was as follows: 33.8% B2, 28.4% D, 19.6% A, and 18.2% B1 [28]. In our study, we included patients with acute pyelonephritis who needed hospitalization, including cases of community-acquired, healthcare-associated, and nosocomial infections. The distributions of category of infection in this study may have influenced the phylogenetic groups. Analysis of phylogenetic groups including healthcare-associated UTI and nosocomial UTI of uropathogenic *E. coli* has been rare. Like the results of phylogenetic group analysis of community-acquired UTI in Korea, in this study phylogenetic group B2 was the most common in community-acquired acute pyelonephritis (APN), healthcare-associated APN and nosocomial APN [24]. In addition, recurrent UTI accounted for 11.6% in phylogenetic group B2 and 9.7% in group D, which should be considered when setting the vaccine target.

Uropathogenic *E. coli* have virulence factors, such as adhesion molecules, toxins, iron acquisition, immune evasion, and protectins [35]. In this study, virulence factors related to adhesion, iron metabolism, and protection were identified in both phylogenetic groups B2 and D. *FimH*, an adhesion molecule-associated virulence factor and *fyuA*, an iron metabolism-related virulence factor were the most and second most frequently detected virulence factors. The virulence factors that exhibited differences in distributions between the two groups were type I *fimbriae; focG, sfa/focED* in adhesion molecules; *hlyA, cnf1* in toxins; *fyuA* in iron metabolism; and *PAI.* Adhesion molecules, such as type I *fimbriae*, play an important role in the attachment of *E. coli* to the mucosal epithelium, initiation of biofilm formation, and persistence in the bladder [17]. In a comparative study of UTIs with and without bacteremia in Sweden, adhesion molecules such as *papG (P fimbriae)* were more frequently observed in bacteremic UTI than in non-bacteremic UTI [36]. In a study of UTI at outpatient clinics, risk factor analysis of virulence factors affecting phylogenetic groups revealed that strains with *papC* and *sfa* genes were associated with the phylogenetic group B2 [26]. In addition, biofilm formation in *E. coli* was observed in strains harboring adhesion-associated virulence genes [37]. Toxin-related virulence factors are important for mediating bacterial invasion and for the dissemination and persistence of bacteria in the bladder [38–40]. *HlyA* is needed for initial bacterial invasion, and *cnf1* is needed for bacteria dissemination and persistence [16, 39–41]. In a UTI-infected mouse model, *hlyA* accelerated bacteremia to fulminant sepsis [41]. *HlyA*-expressing uropathogenic *E. coli* activated caspase-independent necroptosis, but not caspase-mediated apoptotic cell death, and the products released from damaged cells by necroptosis induced proinflammatory response in macrophages [39]. In *cnf1*- and *hlyA*-expressing uropathogenic *E. coli*, higher urinary levels of proinflammatory cytokines were detected than in pathogens not expressing such virulence factors [40]. Iron uptake systems and siderophores facilitate iron scavenging in the environment [16]. In a study of *E. coli* bacteremia in Spain, strains expressing *fyuA* were associated with increased mortality during hospital stay [42]. *FyuA* causes invasion of bacteria into the bloodstream from the urinary tract and is associated with highly pathogenic strains [19, 42]. Various vaccines are being developed according to the mechanisms of virulence factors, mainly targeting adhesion molecules and iron metabolism [19, 22]. Vaccines related to toxins have not yet achieved significant results [13].

The phylogenetic group B2 has been associated with high antimicrobial resistance rates [26, 43]; this may have been influenced by a combination of several factors [44]. Several studies have reported that biofilm formation is associated with a high antibiotic resistance rate [34, 45]. Multiple virulence factors, such as *α-hemolysin*, lipopolysaccharides, proteases, adhesins, aerobactin, and fimbriae significantly affect biofilm formation [16]. The phylogenetic group B2 was associated with adhesion molecules and biofilm formation to a greater degree than other phylogenetic groups [45]. Drug resistance in uropathogenic *E. coli* strains is more likely caused by biofilm formation, and the biofilms have potential roles in recurrent infections and antibiotic resistance [34, 45, 46]. Several studies have reported virulence factors associated with the antimicrobial resistance of uropathogenic *E. coli* [25, 26, 34, 47]. PAI is also associated with antimicrobial resistance [26]. In a study of symptomatic UTIs in outpatients in Iran, *hlyA, malX*, and *hlyA* were revealed as risk factors among virulence factors affecting antimicrobial resistance to ciprofloxacin and ceftriaxone [34]. Another study of UTIs including cystitis and pyelonephritis in Turkey showed that *afa/draC* and *iha* were the virulence factors associated with antimicrobial resistance [25]*.* In phylogenetic group D, among underlying diseases, diabetes mellitus was more common than in phylogenetic group B, and the frequency of bacteremia was also higher. Dysregulated immune pathways in diabetes mellitus contribute to the impairment of host responses in sepsis. Diabetic patients were more likely to develop acute kidney injury [48]. Although the strains of phylogenetic group B were more virulent than the strains of phylogenetic group D, there was no difference in clinical outcomes between the two groups, possibly due to differences in the frequency of underlying diseases, especially diabetes mellitus. Further research will be needed to determine whether there are other factors besides the underlying disease that are not significantly different from the clinical outcomes of phylogenetic groups B2 and D.

There are several limitations to this study. First, this study was retrospective; therefore, we had to rely on the medical records, and it was difficult to evaluate urinary function and identify the subjective urinary symptoms in all patients. Second, we acknowledge that the patients included in this study were at a tertiary hospital, and their condition might have been more severe than that of patients in a primary medical center. Despite these limitations, we found differences in the virulence factors, antimicrobial susceptibility, and clinical presentations of uropathogenic *E. coli* according to the phylogenetic group.

## Conclusion

In conclusion, in cases of pyelonephritis with uropathogenic *E. coli*, we observed differences in the virulence factors and antimicrobial resistance rates between phylogenetic groups B2 and D. Further studies will be needed to elucidate the virulence factors of uropathogenic *E. coli* according to phylogenetic group and host interaction. As differences in genetic and phenotypic characteristics occur based on strains, various therapeutic options targeting virulence factors may be considered along with antibiotics.

## Data Availability

The dataset of the current study are available from the corresponding author upon request.

## Abbreviations

UPEC: Uropathogenic *Escherichia coli*
UTI: Urinary tract infection
PCR: Polymerase chain reaction
DNA: Deoxyribonucleic acid
ESBL: Extended spectrum beta-lactamase
CLSI: Clinical and Laboratory Standards Institute
DM: Diabetes mellitus
APN: Acute pyelonephritis
min: Minute
